# *Lactobacillus rhamnosus* GG ameliorates noise-induced cognitive deficits and systemic inflammation in rats by modulating the gut-brain axis

**DOI:** 10.3389/fcimb.2023.1067367

**Published:** 2023-04-26

**Authors:** Xiaofang Li, Pengfang Zheng, Wa Cao, Yang Cao, Xiaojun She, Honglian Yang, Kefeng Ma, Fangshan Wu, Xiujie Gao, Yu Fu, Jiayi Yin, Fei Wei, Shoufang Jiang, Bo Cui

**Affiliations:** ^1^ Tianjin Institute of Environmental and Operational Medicine, Tianjin, China; ^2^ School of Public Health and Management, Binzhou Medical University, Yantai, China; ^3^ School of Public Health and Management, Weifang Medical University, Weifang, China; ^4^ School of Public Health, North China University of Science and Technology, Tangshan, China; ^5^ Shandong Academy of Occupational Health and Occupational Medicine, Shandong First Medical University, Jinan, China

**Keywords:** noise, *Lactobacillus rhamnosus* GG, cognition, inflammation, gut microbiota, gut-brain axis

## Abstract

**Background:**

Environmental noise exposure is linked to neuroinflammation and imbalance of the gut microbiota. Promoting gut microbiota homeostasis may be a key factor in relieving the deleterious non-auditory effects of noise. This study aimed to investigate the effect of *Lactobacillus rhamnosus* GG (LGG) intervention on noise-induced cognitive deficits and systemic inflammation in rats.

**Methods:**

Learning and memory were assessed using the Morris water maze, while 16S rRNA sequencing and gas chromatography-mass spectrometry were used to analyze the gut microbiota and short-chain fatty acid (SCFA) content. Endothelial tight junction proteins and serum inflammatory mediators were assessed to explore the underlying pathological mechanisms.

**Results:**

The results indicated that *Lactobacillus rhamnosus* GG intervention ameliorated noise-induced memory deterioration, promoted the proliferation of beneficial bacteria, inhibited the growth of harmful bacteria, improved dysregulation of SCFA-producing bacteria, and regulated SCFA levels. Mechanistically, noise exposure led to a decrease in tight junction proteins in the gut and hippocampus and an increase in serum inflammatory mediators, which were significantly alleviated by *Lactobacillus rhamnosus* GG intervention.

**Conclusion:**

Taken together, *Lactobacillus rhamnosus* GG intervention reduced gut bacterial translocation, restored gut and blood-brain barrier functions, and improved gut bacterial balance in rats exposed to chronic noise, thereby protecting against cognitive deficits and systemic inflammation by modulating the gut-brain axis.

## 1 Introduction

The intestine has the most abundant and diverse bacterial community in the body (Sender et al., 2016). The gut microbiota, known as the “second brain”, affects normal physiology, synaptic, immune, and barrier functions, and host behavior, including cognition, through the microbiome-gut-brain axis (Shukla et al., 2021). Dysbiosis of the gut microbiota frequently leads to brain (Hill-Burns et al., 2017; Pulikkan et al., 2018; Singhrao and Harding, 2020) and gut diseases, such as inflammatory gut disease (Al Bander et al., 2020). Our previous study demonstrated that noise intervention changed the gut microbiota, increased gut and brain endothelial barrier dysfunction, and accelerated neurochemical and inflammatory dysregulation in an Alzheimer’s disease (AD) mouse model (Cui et al., 2018). Moreover, noise exposure leads to changes in the relative abundance of species belonging to the family *Lactobacillaceae* (including the genus *Lactobacillus*) (Chi et al., 2021), although other factors may also cause *Lactobacillus* disorders. For instance, the abundance of *Lactobacillus* is reduced in mice with high-fat diet induced steatohepatitis (Zhou et al., 2017).

*Lactobacillus Rhamnosus* GG (LGG) is a probiotic originally isolated from the human gut. In recent years, studies have shown that LGG can tolerate the environment of the digestive tract and colonize the gut, participating in the regulation of intestinal microbiota homeostasis (Ritze et al., 2014; Chen et al., 2019; Zhao et al., 2020).Sanborn et al. (Sanborn et al., 2020) reported that cognitive performance improved in older adults after supplementation with probiotic LGG, suggesting that LGG intervention may delay aging-related cognitive decline. Additionally, supplementation with *Lactobacillus* and *Bifidobacterium* reportedly improved spatial learning, memory deficits, and oxidative stress in AD rats (Athari Nik Azm et al., 2018). Moreover, LGG plays a key role in protecting against inflammatory injury and intestinal barrier dysfunction (Donato et al., 2010). For example, LGG reportedly regulates the expression of tight junction proteins Occludin and zonula occludens-1 to alleviate impaired barrier function (Han et al., 2019). Notably, one study demonstrated that LGG colonization in early life reduced inflammation, increased the abundance of SCFA-producing bacteria, and promoted the production of SCFAs in young mice, with the beneficial effects persisting up to eight months (Liu et al., 2021).

The above studies suggest potential beneficial effects of LGG on cognitive function, systemic inflammation, intestinal barrier function, and gut metabolism. Thus, the purpose of this research was to investigate whether intervention with LGG may improve noise-induced cognitive deficits and systemic inflammation by modulating the gut-brain axis in rats exposed to chronic noise.

## 2 Materials and methods

### 2.1 LGG culture

0.5 mL of sterile water was injected into LGG lyophilized powder (BeiNa Chuanglian Biotechnology Co., LTD.), gently blown, and fully dissolved into the bacterial suspension. The bacterial suspension was absorbed and 200 μL was added to two MRS solid mediums (Solarbio Science and Technology Co., Ltd. Beijing), evenly spread, and incubated at 37°C for 24 h in an anaerobic station with a gas-pak. LGG single colonies were then selected and added to MRS liquid medium (pH 6.2 ± 0.2, autoclaved at 121°C for 15 min) and incubated at 37°C for 24 h in an anaerobic station with a gas-pak. Subsequently, the bacteria were centrifuged at 4000 × *g* for 15 min at 4°C and the bacterial pellet was collected. The bacteria were resuspended at a concentration of 1 × 10^8^ CFU/mL after the precipitate was washed with saline solution 3 times. The bacterial cultures were stored at 4°C for a short time (within 30 min) during the treatment of the samples.

### 2.2 Animals and experimental groups

A total of 48 healthy, male, six-week-old Wistar rats were provided by Beijing Viton Lihua Laboratory Animal Technology Co., Ltd. (Beijing, China) (animal quality certificate: 2016-0006). The rats were maintained under standard housing conditions with an ambient temperature of 23 ± 2°C and 50%–60% humidity. The rats were acclimated for five days prior to the experiment. Rats were fed a standard laboratory rodent diet and had free access to water (diet and water were autoclaved prior to ingestion). The rats were randomly assigned to the following groups: Control, LGG, Noise, and Noise + LGG. The Control and Noise groups received 1 mL saline solution by gavage daily. The LGG and Noise + LGG groups received 1 mL of LGG suspension daily by gavage. For 56 days, the Noise and Noise + LGG groups were exposed to 88 dB sound pressure level (SPL) white noise for 4 h/day, whereas the Control and LGG groups were exposed to background noise (< 40 dB SPL). All animal experimental protocols were approved by the Tianjin Institute of Environmental and Operational Medicine Animal Use and Research Committee (approval number: LACUC of AMMS-04-2020-063).

### 2.3 Noise exposure set-up

Noise was produced by a noise generator (BK 3560 C, Brüel & Kjær Instruments, Nærum, Denmark), which was then amplified by a power amplifier and broadcast *via* a loudspeaker. The frequency range of the generator’s noise signal was 20-20,000 Hz. In a reverberation chamber, the rats were housed in wire mesh cages in the center of the sound field and exposed to the noise *via* the loudspeaker hung above the cage.

### 2.4 Morris water maze testing

The Morris water maze test adapted from a previous study (Chi et al., 2021), using hidden platform training (spatial learning) and a probe trial (spatial memory). During the platform training phase, the rats sought a hidden platform 2 cm below the water surface. The rats were set in one of the four quadrants of the pool, facing the wall (alternating clockwise in each trial), and were allowed to stay on the platform for 10 s after finding it. If the rats did not find the platform within 60 s, they were manually placed on the platform for 10 s. For four days in a row, the rats finished four trials per day. On day 5 during the probe trial session, the platform was removed, and each rat was allowed to swim freely for 60 s. Video cameras were used to record the movements of the rat and to obtain the target quadrant distance/total distance, number of crossings over the target quadrant, time spent in the target quadrant during training sessions, and escape latency.

### 2.5 Sample collection

After noise exposure, the rats were weighed and anesthetized by intraperitoneal injection of 10% chloral hydrate at a dose of 0.3 mL/100 g. After the abdominal aorta blood collection, the rats were immediately executed by decapitation. Colon tissue was cut with sterile scissors, and colon feces were collected with sterile tweezers in sterile cryopreserved tube, hippocampus and colon tissues were collected and stored in sterile tubes, which were immediately frozen with liquid nitrogen and stored in a −80°C refrigerator for later use.

### 2.6 Transmission electron microscopy and hematoxylin-eosin staining of colon tissue

A sample of colon tissue (1–2 mm^3^) was excised from each rat, to which 2.5% glutaraldehyde was added, and stored at 4°C. Tissues were fixed with osmium acid, dehydrated using an alcohol gradient, permeabilized with encapsulant epoxy, polymerized at 60°C for 48 h, and then stored at room temperature for approximately 20 days. Ultrathin sections (50 nm) were sliced, double stained with uranyl acetate and lead citrate at room temperature for 15 min, dried at room temperature overnight, and observed using transmission electron microscopy.

Additionally, a sample of colon tissue was fixed with 4% paraformaldehyde solution, dehydrated using an ethanol gradient, rendered transparent with xylene, embedded in paraffin, cooled to -20°C, and sliced into sections (5 μm). Subsequently, sections were heated at 45°C in a water bath, gently flattened, and dried at 60°C. After staining with HE (Fan et al., 2019), the sections were rendered transparent with xylene for 10 min, sealed with neutral resin, and observed under an optical microscope.

### 2.7 Western blot analysis

Total proteins of colonic and hippocampal tissues were extracted with RIPA buffer containing protease inhibitors (Solarbio, Beijing, China). The colon and hippocampus were treated with ultrasound for 1 min in a high throughput tissue grinder. The tissue lapping solution was centrifuged at 4°C at 12,000 rpm for 10 min, and the obtained supernatant was used for further analysis. Western blotting analysis was conducted using standard procedures, employing rabbit anti-Occludin (1:1000, Bioworld, USA), rabbit anti-CLDN1 (1:2000, Bioworld, USA), and rat anti-GAPDH (1:10,000, Bioworld, USA) antibodies, and GADPH as an internal reference standard.

### 2.8 Enzyme-linked immunosorbent assay

Serum was obtained by centrifuging abdominal aorta blood for 10 min at 3000 × *g*. The serum was stored at −80°C until analysis *via* ELISA. The following ELISA kits were used: Rat β-amyloid peptides (Aβ) 1-40 ELISA kit (Jiancheng Bioengineering, China), Rat Aβ1-42 ELISA kit (Jiancheng Bioengineering, China), Rat nuclear factor-kappa B (NF-κB) ELISA kit (Thermo Fisher Scientific, USA), Rat interleukin (IL)-10 ELISA kit (Thermo Fisher Scientific, USA), Rat IL-17 ELISA kit (Thermo Fisher Scientific, USA), Rat D-lactic acid (D-LA) ELISA kit (Thermo Fisher Scientific, USA), and Rat lipopolysaccharide (LPS) ELISA kit (Thermo Fisher Scientific, USA).

### 2.9 Sequencing of 16S ribosomal RNA genes in microbiota

Amplicon sequencing was employed to sequence the 16S rRNA genes of the microbiota, as previously described (Chi et al., 2021). Briefly, microbial DNA was isolated from the colon contents using the cetyltrimethylammonium bromide (CTAB) method. An appropriate amount of DNA was diluted to 1 ng/μL in sterile water. Next, the 16S rRNA V4 region were amplified using specific primers with the barcode (16S V4: 515F-806R; 515F 5’-GTGCCAGCMGCCGCGGTAA-3’, 806R 5’-GGACTACHVGGGTWTCTAAT-3’). PCR reactions were performed using Phusion^®^ High-Fidelity PCR Master Mix (New England Biolabs, Ipswich, MA, USA). PCR products were detected by electrophoresis with 2% agarose gel. Additionally, PCR products were mixed in equidensity ratios and purified with the Qiagen Gel Extraction Kit (Qiagen, Germany). The TruSeq^®^ DNA PCR-free Sample Preparation Kit was then employed to construct the library, which was quantified by Qubit and Q-PCR. NovaSeq6000 was used for on-machine sequencing. Paired-end reads were assigned to samples based on their unique barcodes and were truncated by removing the barcode and primer sequences. FLASH (V1.2.7, http://ccb.jhu.edu/software/FLASH/) was then used to merge paired-end reads. This program merges paired-end reads when a portion of the reads overlap with the read generated from the opposite end of the same DNA fragment. The spliced sequences were designated raw tags, which were quality filtered under specific filtering conditions to obtain high-quality clean tags according to the QIIME (V1.9.1, http://qiime.org/scripts/split_libraries_fastq.html) quality control process. Chimera sequences were then detected, and subsequently removed, by comparing the tags with the reference database (Silva database, https://www.arb-silva.de/) using the UCHIME algorithm (UCHIME Algorithm, http://www.drive5.com/usearch/manual/uchime_algo.html). The effective tags were obtained using Uparse software (Uparse v7.0.1001, http://www.drive5.com/uparse/); sequences with ≥ 97% similarity were assigned to the same operational taxonomic unit (OTU). Representative OTU sequences were selected for further annotation.

Alpha and beta diversity analyses were conducted using the QIIME software (Version 1.9.1) and R software (Version 2.15.3), whereas the principal coordinate analysis (PCoA) analysis was performed using weighted UniFrac. Additionally, linear discriminant analysis effect size (LEfSe) analysis was completed with the LEfSe software using a linear discriminant analysis (LDA) score cut off of > 4.0, *p* < 0.05. Analysis of similarity (ANOSIM) was performed with the Rvegan’s Anosim function. Spearman correlation analysis was used to assess the correlation between intestinal microflora and fecal SCFAs. Analysis was performed using Novogene Bioinformatics Technology Co., Ltd. (Beijing, China).

### 2.10 Gas chromatography-mass spectrometer analysis

A total of 50 mg of the fecal sample was homogenized with 50 μL of 15% phosphoric acid, 125 μg/mL of internal standard (isohexanoic acid) solution, 100 μL and 400 μL of ether for 1 min and centrifuged at 12,000 rpm at 4°C for 10 min. The supernatant was then collected for testing. GC-MS was performed using a TRACE™ 1310 ISQ LT GC-MS (Thermo Fisher Scientific, Waltham, MA, USA) with an HP-INNOWAX GC column (30 m × 0.25 mm ID × 0.25 μm; Agilent Technologies, Santa Clara, CA, USA) under the following conditions: sample volume, 1 μL; split ratio, 10:1; inlet temperature, 250°C; ion source temperature, 230°C; transmission line temperature, 250°C; quadrupole temperature, 150°C. The initial oven temperature was 90°C, which was increased to 120°C with a ramp rate of 10°C/min, then to 150°C with a ramp rate of 5°C/min, and finally increased to 250°C with a ramp rate of 25°C/min, and was held at 250°C for 2 min. Helium was used as the carrier gas at a flow rate of 1.0 mL/min. We used Thermo Chromeleon 7.2.10 (Novogene Bioinformatics Technology Co., Ltd.) to process the raw data coming out of the machine. The SCFA concentration was calculated from the respective peak areas of the sample and the internal standard, isohexanoic acid. The construction range and related information of calibration curves for GC-MS are provided in **Supplementary Table 1**.

### 2.11 Statistical analysis

The data are presented as the mean ± standard deviation. Statistical analysis was performed using IBM SPSS Statistics v.22.0 and GraphPad Prism v.7 software. Data were analyzed using one-way analysis of variance (ANOVA), and differences were considered statistically significant when *p* < 0.05. The Kruskal–Wallis test was used when data were not normally distributed. For two-way comparisons between groups, the least significant difference test was used if the variances were equal, and the Games-Howell test was used if they were not. Correlations were identified using Spearman’s rank correlation analysis.

## 3 Results

### 3.1 Effect of LGG intervention on cognitive impairment after noise exposure

**Figure 1:**
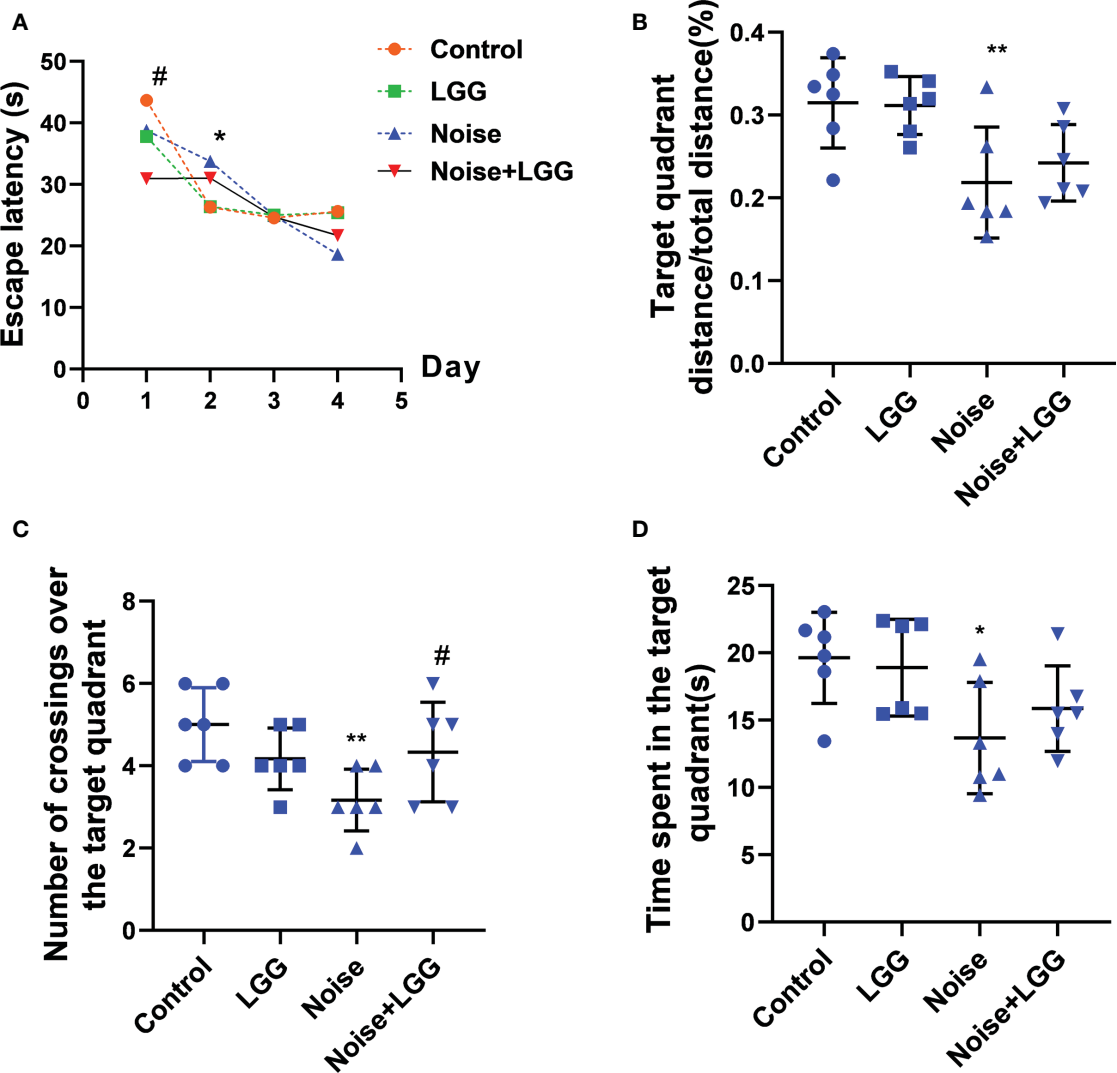
Effects of LGG intervention improved learning and memory abilities in rats. **(A)** Effects of noise exposure on escape latency in the training phase; **(B)** Effects of noise exposure on target quadrant distance/total distance in the probe trial; **(C)** Effects of noise exposure on number of crossings over the target quadrant in the probe trial; **(D)** Effects of noise exposure on time spent in the target quadrant in the probe trial. *n* = 6; *p < 0.05 and **p < 0.01 represent the comparison between Control and Noise group; #*p* < 0.05 represent the comparison between Noise and Noise + LGG group.

To assess changes in cognitive ability, rats underwent Morris water maze testing after noise exposure for 56 days. The escape latency of rats in the Noise group was prolonged on the second day of training compared to that of rats in the Control group (*p* < 0.05). Rats in the Noise + LGG group showed a statistically significant (*p* < 0.05) decrease in escape latency on the first day of training compared with rats in the Noise group (**Figure 1A**). Additionally, the time spent in the target quadrant, number of platform crossings, and target quadrant distance/total distance were reduced in the Noise group (**Figures 1B–D**), and the number of traverses in the target quadrant and time spent in the target quadrant were increased in the Noise + LGG group (**Figures 1C, D**). These results showed that LGG intervention had a protective effect against noise-induced cognitive impairment in rats.

### 3.2 Effect of LGG intervention on gut microbiota after noise exposure

The composition of the gut microbiota in each treatment group was detected *via* 16S rRNA high-throughput sequencing, and gut microbiota species richness (α-diversity) was reflected by Chao1 and ACE indices. The Chao1 and ACE indices were elevated in the Noise group (**Supplementary Figures S1A, B**), indicating that species richness of the gut microbiota in rats was disordered after noise exposure. Comparatively, the Chao1 and ACE indices were reduced in the Noise + LGG group, indicating that LGG intervention could regulate the gut microbiota in rats towards homeostasis. β diversity was obviously decreased after noise exposure compared with the control group (Tukey’s test, *p* < 0.05; **Supplementary Figure S1C**). However, β diversity in the Noise + LGG group was significantly increased (*p* < 0.05) compared with the Noise group, indicating that LGG intervention could regulate both the richness and variety of the gut microbiota in rats.

The variation, confirmed by PCoA and ANOSIM analyses, was higher between groups than within groups (**Supplementary Figures S1D, F, G**). The distribution of the microbiota of the LGG group was relatively concentrated, while that of the Noise group was relatively dispersed, indicating that noise exposure and LGG intervention affected the abundance and structure of the gut microbiota. Based on the species annotation results, the top 10 most abundant bacteria of each treatment group were selected for clustering analysis based on the weighted UniFrac distance matrix (unweighted pair group method with arithmetic mean; UPGMA). Subsequently, the UPGMA results were combined with the relative abundance of the gut microbiota in each treatment group (**Supplementary Figure S1E**). *Firmicutes*, *Bacteroidetes*, and *Proteobacteria* were the most abundant phyla in all treatment groups. Compared to the Control group, the relative abundance of *Bacteroidetes* was increased while that of *Firmicutes* was decreased in the Noise group. Meanwhile, the abundance of *Firmicutes* was increased while that of *Bacteroidetes* and *Proteobacteria* was decreased after LGG intervention compared with the Noise group.

**Figure 2:**
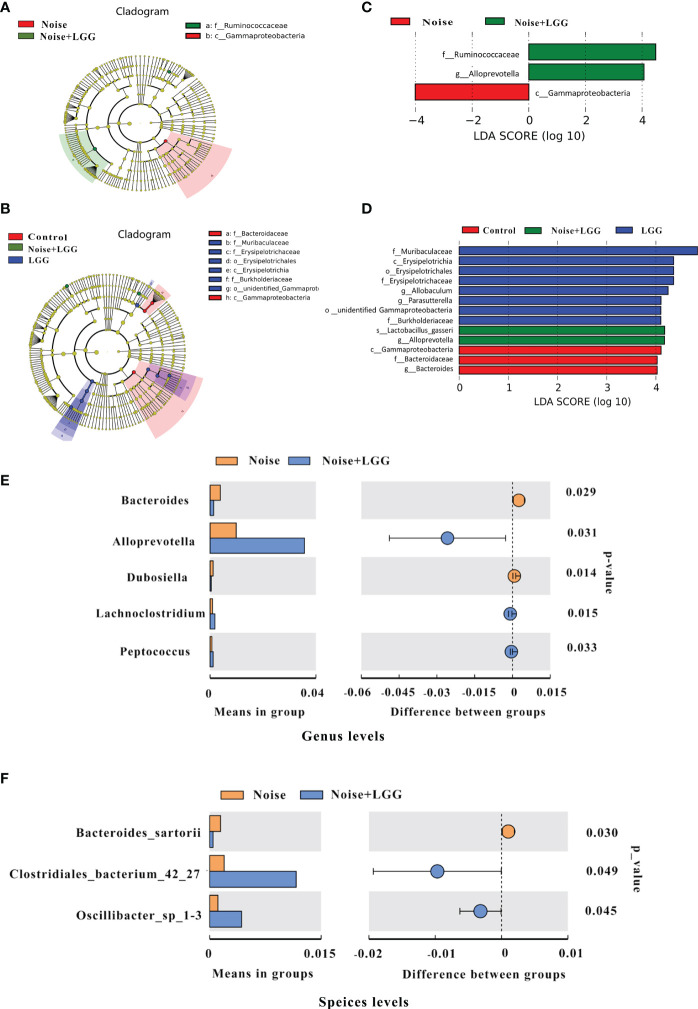
LGG regulates the composition of gut microbiota. **(A, B)** Cladogram based on linear discriminant analysis effect size (LEfSe) analysis. The central point represents the root of the tree (bacteria), and each ring represents the next lower taxonomic level (phylum to genus). The diameter of each circle represents the relative abundance of the taxon (*n* = 10–11). **(C, D)** The most differentially abundant taxa in each group identified by linear discriminant analysis (LDA) scores generated from the LEfSe analysis (*n* = 10–11). **(E, F)** Relative abundances, P-values, and confidence intervals of the gut microbial community at the genus and species level in the Noise group and Noise + LGG group (T-test analysis, *p* < 0.05, *n* = 10–11).

In order to further identify differences in the gut microbiota between the four treatment groups, changes in the composition of the gut microbiota were further assessed using the LEfSe test, and the dominant flora in each group were represented by cladograms (**Figures 2A, B**). The taxa with the greatest differences from phylum to genus were identified using LDA scoring (**Figures 2C, D**). The dominant species in the Control group belonged to the *Bacteroidaceae* and *Gammaproteobacteria*, while those in the Noise group belonged to the *Gammaproteobacteria*. The dominant species in the LGG group belonged to the *Muribaculaceae*, *Erysipelotrichaceae*, and *Burkholderiacea*, and the genera *Allobaculum* and *Parasutterella*, while those in the Noise + LGG group included *Lactobacillus gasseri* and species belonging to the *Ruminococcaceae* and *Alloprevotella*. Furthermore, at the class and family levels, LEfSe analysis showed that the abundance of *Gammaproteobacteria* in the Noise + LGG group was decreased compared with that in the Noise group. While the species abundances of *Lactobacillus gasseri*, which belongs to *Lactobacillaceae* and *Ruminococcaceae*, increased (**Supplementary Figures S2A–C**).

To clearly demonstrate the effect of LGG intervention on the regulation of intestinal microbiota after noise exposure, we used T-test to analyze the differences in microbiota between Noise and Noise + LGG groups at the genus level and the species level, respectively. Compared with the Noise group, the relative abundances of *Alloprevotella*, *Lachnoclostridium*, and *Peptococcus* increased significantly in the Noise + LGG group at genus level after LGG treatment; whereas the abundances of *Bacteroides* and *Dubosiella* decreased significantly in the Noise + LGG group (*p* < 0.05; **Figure 2E**). Similarly, at species level, the relative abundances of *Clostridiales_bacterium_42_27* and *Oscillibacter_sp_1-3* increased more in the Noise + LGG group than Noise group, while the *Bacteroides sartorii* decreased (*p* < 0.05; **Figure 2F**). In addition, Tax4fun analysis was used to predict functional differences of gut microbiota between groups, and the results are supplemented in the **Supplementary Figure S3**.

### 3.3 Changes of SCFA levels and metabolites in gut microbiota after LGG intervention

**Figure 3:**
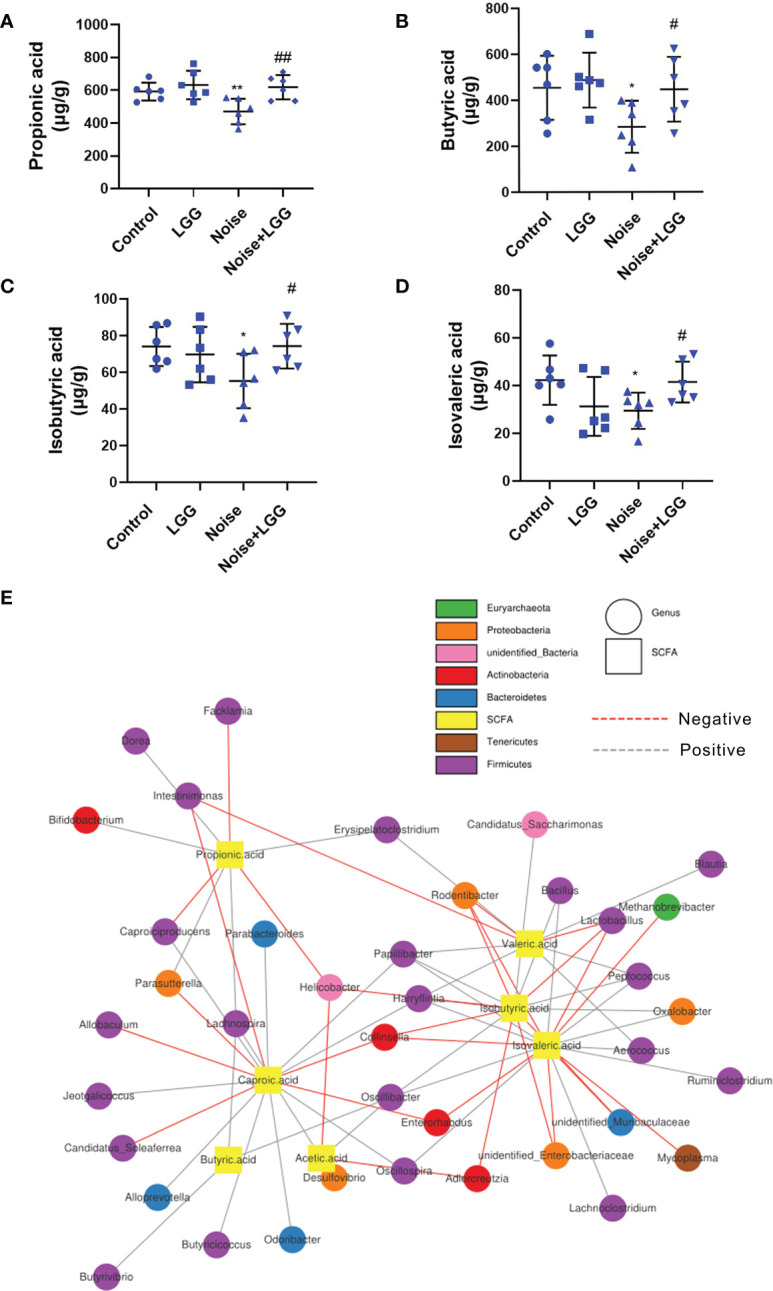
Analysis of the level of SCFAs and its correlation with gut microbiota. LGG intervention reversed the reduction in SCFAs levels caused by noise exposure **(A–D)**. **(A)** Propionic, **(B)** Butyric, **(C)** Isobutyric, and **(D)** Isovaleric acids. *n* = 6; *p < 0.05 and **p < 0.01 represent the comparison between Control and Noise group; #*p* < 0.05 and ##*p* < 0.01 represent the comparison between Noise and Noise + LGG group. **(E)** Network Diagram was used to show the associations between the SCFAs metabolites and genus levels of gut microbiota.

SCFA levels in the feces of rats in each treatment group are shown in (**Figures 3A–D**). Levels of propionic acid, butyric acid, isobutyric acid, and isovaleric acid were reduced in the Noise group compared to those in the Control group, whereas levels of these SCFAs were increased after LGG intervention. These results indicated that LGG intervention inhibited the noise-induced decrease in SCFAs levels in rats.

Microbial metabolites are one of the main communication channels for crosstalk between bacteria and hosts. As the main product of bacterial fermentation, changes in SCFAs content alter the acidic environment in the gut, affect the normal growth of gut microbes, and influence the composition and structure of the gut microbiota. Therefore, correlation analysis was conducted to evaluate the associations between the SCFA metabolites and genus level of gut microbiota. The correlation network diagram provided the visualization of the relationship (**Figure 3E**). For example, butyric acid was positively correlated with *Butyrivibrio* (*r* = 0.393, *p* = 0.011); propionic acid was positively correlated with *Bifidobacterium* (*r* = 0.333, *p* = 0.033), *Lachnospira* (*r* = 0.306, *p* = 0.051), and *Dorea* (*r* = 0.331, *p* = 0.035); isobutyric acid was positively correlated with *Oscillibacter* (*r =* 0.331, *p* = 0.034), *Peptococcus* (*r* = 0.439, *p* = 0.004), and *Bacillus* (*r* = 0.312, *p* = 0.047), while negatively correlated with *Unidentified_Enterobacteriaceae* (*r* = -0.368, *p* = 0.018); isovaleric acid had positive correlations with *Oscillibacter* (*r* = 0.366, *p* = 0.019), while being significantly negatively correlated with *Enterorhabdus* (*r* = -0.321, *p* = 0.041).

### 3.4 Changes in β-amyloid peptides and serum inflammatory cytokines after LGG intervention

**Figure 4:**
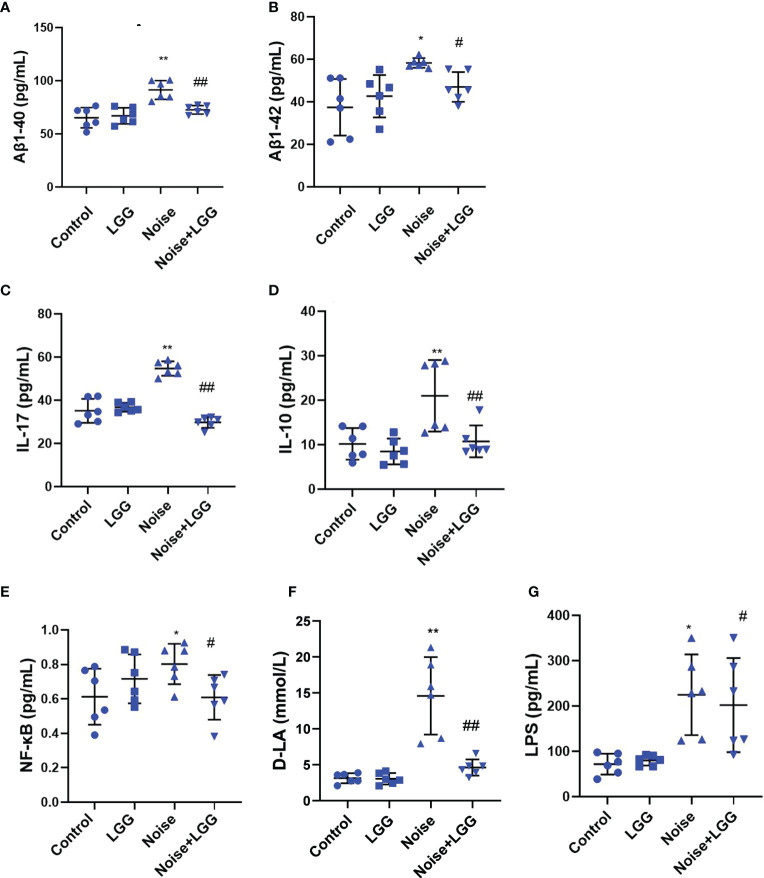
LGG intervention reduces serum inflammatory cytokines and inflammatory markers. **(A, B)** Changes in serum Aβ1-40 and Aβ1-42 in each group. **(C–E)** Changes in serum IL-17, IL-10, and NF-κB in each group. **(F)** Changes in serum D-LA in each group. **(G)** Changes in serum LPS in each group. *n* = 6. *p < 0.05 and **p < 0.01 represent the comparison between Control and Noise group; #*p* < 0.05 and ##*p* < 0.01 represent the comparison between Noise and Noise + LGG group.

Serum levels of Aβ1-40 and Aβ1-42 were higher after noise exposure than those in the Control group (**Figures 4A, B**). However, serum levels of Aβ1-40 and Aβ1-42 in the Noise + LGG group were notably lower (*p* < 0.05) than those in the Noise group. These results indicated that LGG intervention can regulate abnormal increases in Aβ serum levels. Serum inflammatory cytokine and mediator levels in each treatment group are shown in **Figures 4C–E**. IL-17 and IL-10 are important inflammatory cytokines, and NF-κB is a main regulator of natural immunity and inflammation that can be activated by inflammatory cytokines. Serum levels of IL-17, NF-κB, and IL-10 were higher in the Noise group than in the Control group, but lower in the Noise + LGG group than in the Noise group. Serum levels of inflammation markers D-LA and LPS, which are related to gut mucosal damage, were remarkably higher in the Noise group than those in the Control group (**Figures 4F, G**), suggesting injury to the gut mucosa. However, serum levels of D-LA and LPS were significantly reduced after noise and LGG intervention (**Figures 4F, G**). These results showed that LGG intervention may inhibit the release of inflammatory cytokines into the blood and reverse the inflammation caused by noise exposure in rats, thus playing a protective role.

### 3.5 Effect of LGG intervention on epithelial barrier function after noise exposure

**Figure 5:**
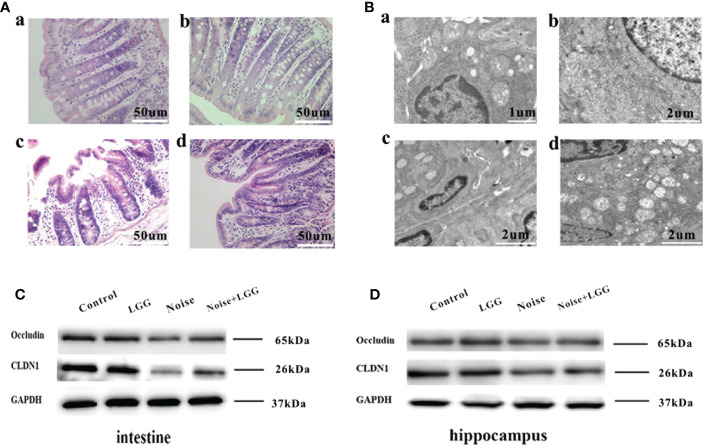
Protective effect of LGG intervention on gut and blood-brain barrier in rats. **(A)** Histopathological changes in HE-stained rat colon sections (magnification ×40). (a) Control, (b) LGG, (c) Noise, and (d) Noise + LGG groups. **(B)** The ultrastructural colon changes were observed with transmission electron microscopy (magnification: a: × 7000, b, c, d: × 5000). (a) Control, (b) LGG, (c) Noise, and (d) Noise + LGG groups. **(C)** The protein levels of occludin and CLDN1 in the colon tissues of rats. **(D)** The protein levels of Occludin and CLDN1 in the hippocampal tissues of rats. GADPH served as an internal reference standard for normalization.

Histopathological analysis (**Figure 5A**) of colon tissues from each treatment group revealed complete mucosal structure, orderly arrangement of epithelial cells, and tightly arranged lamina propria glands in the Control and LGG groups. In the Noise group, the mucosal structure of the colon tissue was incomplete, the epithelium was exfoliated, and the lamina propria glands were short and arranged loosely and irregularly. The mucosal structure of the colon tissue in the Noise + LGG group was incomplete and the lamina propria glands were arranged irregularly, but the pathological changes were reduced compared to those in rats exposed to noise alone. The electron microscopy results of colon tissues in each group (**Figure 5B**) showed that the colon epithelial cells in the Control group and the LGG group were normal, with more mitochondria and clear structure. In the Noise group, the junctions of epithelial cells were significantly widened and there were fewer mitochondria, with some vacuolated. However, colonic epithelial cells in Noise + LGG group were relatively normal, with clear mitochondrial structure and some vacuolated.

Analysis of tight junction proteins in the colon (**Figure 5C**) and hippocampus (**Figure 5D**) of each group revealed that the expression levels of Occludin and CLDN1 were decreased in the colon and hippocampus of the Noise group compared with the control group (**Figures 5C, D**), while they were increased in Noise + LGG group when compared with the Noise group. These results suggested that noise exposure led to impaired gut and blood-brain barrier functions in rats, which was ameliorated with LGG intervention.

## 4 Discussion

**Figure 6:**
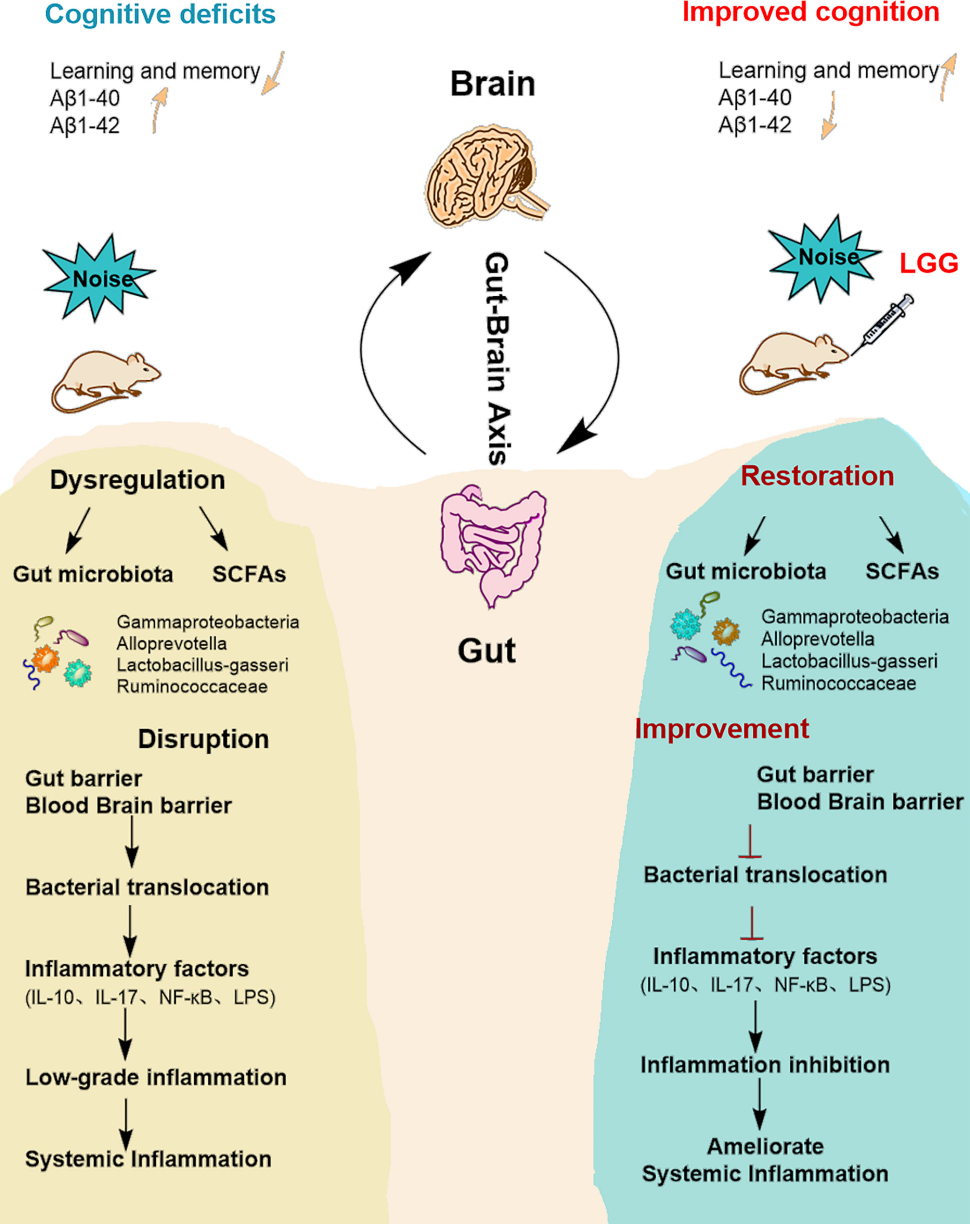
Summarizing central scheme: LGG intervention ameliorates systemic inflammation and cognitive impairment. LGG intervention significantly increased the abundance of beneficial bacteria (*Ruminococcaceae*, *Lactobacillus-gasseri*, and *Alloprevotella*) and decreased harmful strains (*Gammaproteobacteria*). LGG colonization increased the abundance of SCFA-producing bacteria (*Ruminococcaceae*, *Alloprevotella*) and the content of SCFAs. In addition, LGG intervention also improved cognitive function and reduced systemic inflammation through the microbial-gut-brain axis by repairing gut-blood-brain barrier damage, thus avoiding bacterial translocation, reducing inflammatory cytokine levels, and improving learning and memory abilities in rats. LGG, *Lactobacillus rhamnosus* GG; SCFAs, short-chain fatty acids; IL-10, interleukin 10; IL-17, interleukin 17; LPS, lipopolysaccharide.

Our previous studies showed that noise exposure alters the gut microbiota (Cui et al., 2016), inducing oxidative inflammation and AD-like neuropathy (Chi et al., 2021). The results of the current study confirmed that chronic low-intensity noise exposure could induce numerous microbiome-gut-brain axis events. Moreover, the results indicated that LGG intervention could ameliorate noise-induced gut microbiota disturbance, gut and blood-brain barrier dysfunction, cognitive impairment, and systemic inflammation, which may provide new insights into treating the neurological effects of environmental noise exposure (**Figure 6**).

In our previous studies (Cui et al., 2018; Confer et al., 2021) long-term high-intensity noise exposure negatively impacted spatial learning and memory and caused cognitive impairment in rats. Gut microbiota dysbiosis, increased systemic inflammation, and reduced integrity of gut and blood-brain barriers may also be factors in noise-induced impairment of cognitive function *via* the microbiome-gut-brain axis. The results of the current research support that low-intensity noise exposure leads to cognitive decline and concur with two recent models of low-intensity noise exposure (Sun G. et al., 2021; Zhang Y. et al., 2021). Our results indicated that after LGG intervention, the behavioral performance of rats improved, which was consistent with the normal control group, suggesting that cognitive deficits were improved by LGG intervention. Therefore, we hypothesize that the microbiome-gut-brain axis may play a key role in the amelioration of cognitive impairment caused by noise exposure.

Additionally, the results of the current study indicate that noise exposure leads to abnormal changes in the gut microbiota and disrupts normal metabolism of SCFAs, which in turn damages the intestinal barrier, creating a vicious cycle (Lee et al., 2020). LGG intervention can ameliorate gut microbiota imbalance and SCFAs metabolism abnormalities caused by noise. *Gammaproteobacteria* are related to increased intestinal permeability, inflammatory cell proliferation, and secretion of inflammatory mediators, leading to activation of the immune inflammatory response (Shin et al., 2015; Panasevich et al., 2018). In addition to providing energy to the host (Huang et al., 2015), *Ruminococcaceae* are known to have beneficial effects on intestinal barrier function (Leclercq et al., 2014). Further, *Lactobacillus-gasseri*, a symbiotic lactic acid bacterium, can inhibit the NF-κB signaling pathway and increase intestinal barrier integrity (Shiraishi et al., 2021). *Lactobacillus-gasseri* can also regulate gut bacterial dysbiosis, alleviate colonic inflammation, and improve cognitive dysfunction in mice (Yun et al., 2020). Moreover, the relative abundance of *Alloprevotella* has been negatively associated with inflammation (Wang et al., 2020) and may also enhance antioxidant capacity (Wang et al., 2021). In this study, LGG intervention increased the abundance of *Alloprevotella* and probiotic bacteria belonging to the *Firmicutes*, such as *Ruminococcaceae* and *Lactobacillus-gasseri*, which are known to produce SCFAs (Kong et al., 2019; Xu et al., 2021).

We found that LGG intervention could improve *Oscillibacter_SP_1-3* and *Clostridiales_bacterium_42_27*, which belong to *Ruminococcaceae* in the Noise group. The elevation of blood LPS concentration had a strong association with *Bacteroides* (Ding et al., 2020). After LGG intervention, the abundance of *Bacteroides* and *Bacteroides-sartorii* decreased in the Noise + LGG group, which is also consistent with reduced levels of LPS in the serum. The beneficial bacteria *Lachnoclostridium* can inhibit the growth of pathogenic bacteria and regulate intestinal homeostasis (Zhang et al., 2020) and *Peptococcus* can promote butyric acid production. Our results also showed that isobutyric acid was positively correlated with *Peptococcus* (Zhang et al., 2022). In addition, the abundance of species belonging to the *Enterobacteriaceae* (*unidentified_Enterobacteriaceae*) in the *Gammaproteobacteria* was negatively correlated with isobutyric acid. LGG decreased the abundance of harmful *Gammaproteobacteria* caused by noise, and the increase of these harmful bacteria led to the higher abundance of gut microbiota in the noise group. These results show that LGG can mitigate the negative effects of noise by increasing the abundance of *Firmicutes* and reversing the *Firmicutes*/*Bacteroidetes* ratio, which is considered an important indicator of gut microbiota health (Li and Ma, 2020). These variations were followed by beneficial changes in gut microbiota diversity and improved SCFAs levels. The study findings demonstrate that LGG intervention can reshape the gut microbiota structure, increase the SCFAs content, and maintain the normal intestinal microenvironment by enriching beneficial bacteria and inhibiting pathogenic bacteria.

The results of the present research indicate that noise exposure increases gut and blood-brain barrier permeability and systemic inflammatory responses in rats, which is consistent with our previous research (Chi et al., 2021). D-LA is used as a serum marker of intestinal permeability (Zhang H. et al., 2021) and LPS can be used as an indicator of gut microbiota translocation (Luchetti et al., 2021). Gut mucosal inflammation is positively correlated with D-LA and LPS levels, which are also associated with increased gut mucosal permeability (Vitetta et al., 2017). Consistently, noise exposure significantly increased serum levels of LPS and D-LA, pro-inflammatory cytokine IL-17, and inflammatory mediators NF-κB, Aβ1-40, and Aβ1-42. Interestingly, noise exposure led to elevated levels of anti-inflammatory cytokine IL-10. NF-κB can drive the differentiation of monocytes into macrophages, with the M1 type producing pro-inflammatory cytokines and the M2 type producing anti-inflammatory cytokines, such as IL-10 (Sun S. et al., 2021). We hypothesize that gut microbiota dysbiosis after noise exposure and excessive release of LPS activates NF-κB, which in turn drives the differentiation of monocytes into macrophages, with the M1 phenotype leading to a massive release of IL-17, thus causing an inflammatory response. Subsequently, the M2 phenotype produces IL-10 to resist the body’s exposure to inflammation.

LGG can act on the gut epithelium to form a barrier and block the invasion of harmful bacteria in the intestine (Rao and Samak, 2013), regulate the NF-κB signaling pathway, and exert certain anti-inflammatory effects (Hou et al., 2019). Therefore, serum levels of D-LA, LPS, and NF-κB decreased after LGG intervention. The reduced levels of IL-17 and IL-10 may be due to less LPS being released after improvement of intestinal damage, thus downregulating NF-κB expression, inhibiting macrophage differentiation, and balancing inflammatory and anti-inflammatory cytokines. In addition, LGG intervention reduced serum levels of Aβ, preventing abnormal entry of Aβ into the brain through the damaged blood-brain barrier, which can damage neurons, exacerbate neuroinflammation, and lead to cognitive dysfunction (Deane, 2012; Long et al., 2019). These results further suggest that LGG regulates gut microbiota homeostasis and SCFAs levels, inhibits the release of inflammatory cytokines, and protects the intestinal barrier. SCFA can not only enhance the intestinal barrier and local anti-inflammatory effect (Luo et al., 2020), but also directly cross the blood-brain barrier and enter the brain tissue (Den et al., 2020). Intake of SCFA can reduce hippocampal neuroinflammation and neuronal apoptosis by inhibiting NF-κB, and improve cognitive performance (Xiao et al., 2022). Therefore, LGG intervention may prevent local inflammation in the gut, avoiding systemic inflammation induced *via* the gut-brain axis pathway that could impair neurological function and increased SCFA may be involved in the improvement of cognitive function.

This study had two major limitations. First, based on animal ethics and principles of the experiment, partial experiments randomly selected only six samples per group for statistical analysis. Second, due to the limited samples, western blotting results were only assessed to observe trends in expression levels, without statistical analyses being performed. Therefore, it is necessary to increase the sample size and adopt a variety of detection methods in the future experimental design and detection.

## 5 Conclusions

Taken together, the study findings indicate that noise exposure disturbs intestinal microbiota homeostasis, SCFAs metabolism, and upregulates systemic low-grade inflammation, which may be the cause of intestinal and brain epithelial barrier deficiencies. LGG intervention can ameliorate cognitive deficits and systemic inflammation in rats exposed to noise, possibly through linked changes in the microbiome-gut-brain axis. This research advances the current knowledge regarding the etiological signaling pathways participating in negative non-auditory effects of environmental noise.

## Data availability statement

The datasets presented in this study can be found in online repositories. The names of the repository/repositories and accession number(s) can be found below: NCBI SRA, PRJNA890543.

## Ethics statement

The animal study was reviewed and approved by Institutional Animal Use and Care Committee of the Tianjin Institute of Environmental and Occupational Medicine.

## Author contributions

Conceptualization, BC, SJ and FW. XL, PZ and WC shared first authors. Methodology, XL, PZ, WC, YC and XS, software, WC, YC. Validation, SJ and FSW. Formal analysis, HY. Investigation, BC. Resources, KM and FW. Data curation, JY and YF. Writing original draft preparation, WC. Writing review and editing, XL and PZ. Visualization, PZ and XL. Supervision, XG. Project administration, XS. Funding acquisition, BC. All authors contributed to the article and approved the submitted version.
