# Supplementary material for: Lactobacillus rhamnosus GG ameliorates noise-induced cognitive deficits and systemic inflammation in rats by modulating the gut-brain axis
Source: Front Cell Infect Microbiol. 2023 Apr 26;13:1067367. doi: 10.3389/fcimb.2023.1067367 (PMC10169735; doi:10.3389/fcimb.2023.1067367)
Supplement: Supplementary file 1 [file DataSheet_1.pdf]

# *Supplementary Material*

## 1 Supplementary Table1

The concentration series of standard solution of short-chain fatty acid were detected by Gas Chromatography-Mass Spectrometer analysis (GC-MS), and the linearity was investigated by using the concentration of standard substance as abscissa and the ratio of peak area between standard substance and internal standard as ordinate. The obtained linear regression equation of each substance is shown in **Table 1** below. Correlation coefficient r>0.99.

**Supplementary Table 1 Linear regression equation, precision, repeatability and limit of quantification of 7 SCFA standards**

| Serial number | Name | Retention Time (min) | Quantitative ion | Linear equation | Correlation coefficient (r) | Linear range (µg / mL) | Intra-day precision RSD (%) | Daytime precision RSD (%) | Repeatability RSD (%) | Quantitative limit (µg/mL) |
|---|---|---|---|---|---|---|---|---|---|---|
| 1 | Acetic acid | 4.52 | 60 | y=0.034x + 0.0051 | 0.9948 | 0.02-500 | 1.85 | 11.73 | 5.44 | 0.02 |
| 2 | Propionic acid | 5.56 | 74 | y=0.0304x + 0.056 | 0.9959 | 0.02-500 | 1.73 | 5.28 | 4.72 | 0.02 |
| 3 | Isobutyric acid | 5.95 | 73 | y=0.0385x + 0.0098 | 0.996 | 0.02-500 | 1.31 | 4.76 | 5.65 | 0.02 |

| | | | | | | | | | | |
|---|---|---|---|---|---|---|---|---|---|---|
| 4 | Butyric acid | 6.82 | 60 | y=0.088x + 0.0125 | 0.9975 | 0.02-500 | 1.57 | 6.87 | 5.51 | 0.02 |
| 5 | Isovaleric acid | 7.47 | 60 | y=0.0974x + 0.0016 | 0.9964 | 0.02-500 | 1.35 | 6.63 | 7.59 | 0.02 |
| 6 | Pentanoic acid | 8.59 | 60 | y=0.0887x + 4e-04 | 0.991 | 0.02-500 | 1.30 | 5.66 | 7.68 | 0.02 |
| 7 | Caproic acid | 10.11 | 60 | y=0.0699x + 0.0137 | 0.9904 | 0.02-500 | 1.00 | 7.71 | 9.53 | 0.02 |

## 2 Supplementary Figure 1

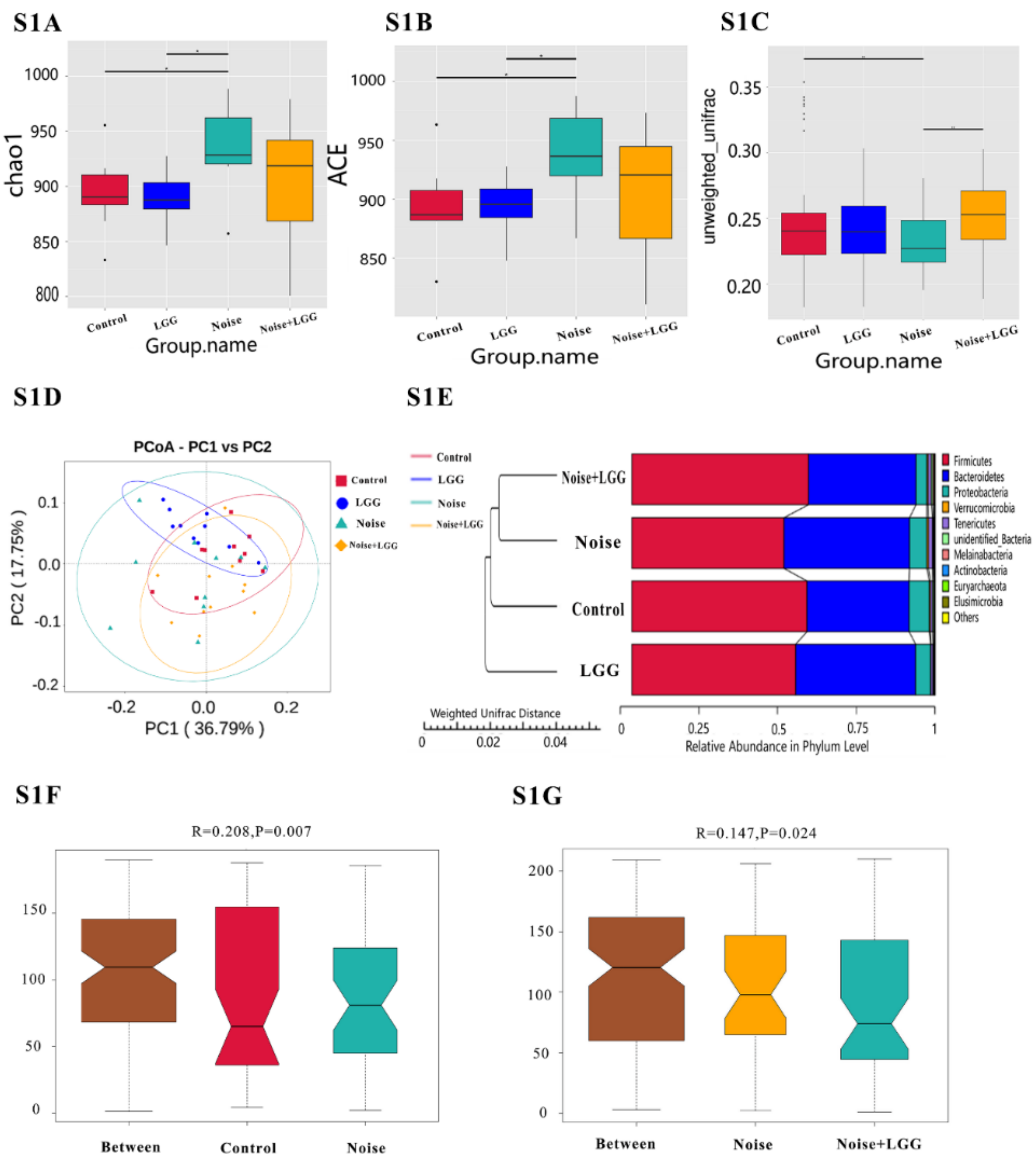


**Supplementary Figure 1. LGG regulates the diversity and abundance of gut microbiota.** (A, B) Chao1 and ACE indices of the observed gut microbial communities in colon content (*n* = 10–11). (C) β diversity analysis of gut microbiota in colon content. (D) PCoA analysis of gut microbial composition in colon content. (E) The composition of gut microbiota in colon content in the different phyla. (F-G) Performed using ANOSIM analysis to assess beta diversity between microbial communities of the two groups.

**Supplementary figure 2**

**A**

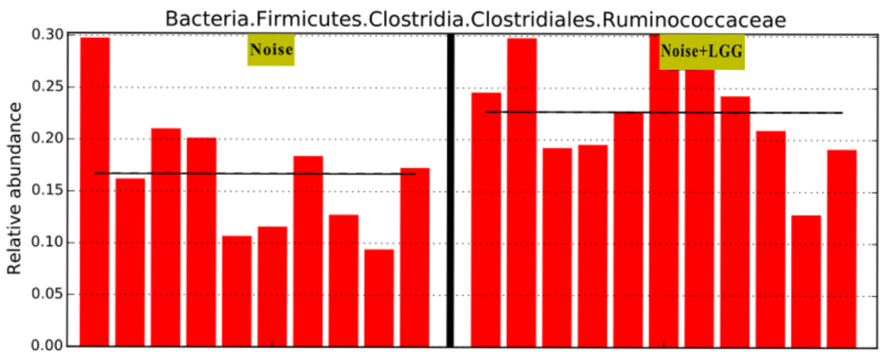


**B**

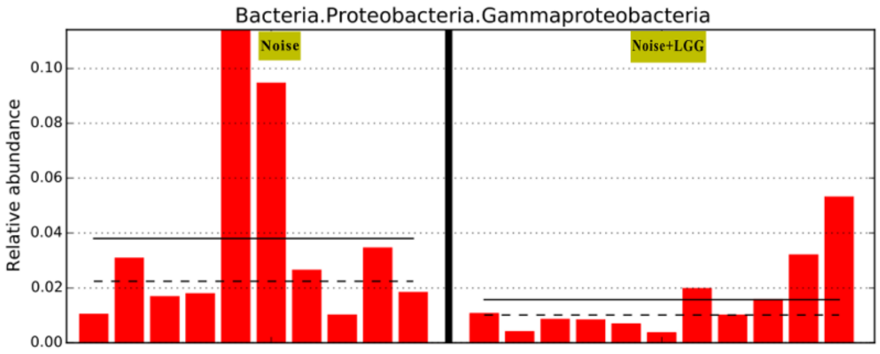


**C**

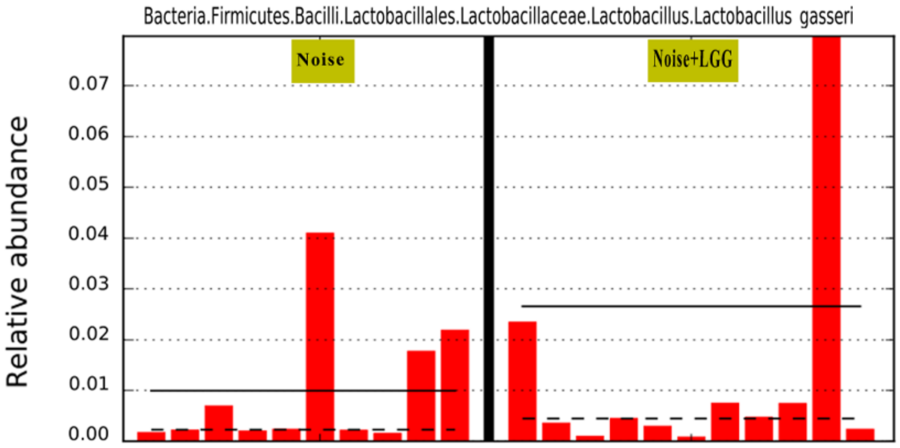


**Supplementary figure 2. LGG regulates the composition of gut microbiota.** (A-C). Changes in the abundance of different microbiota in the Noise and Noise + LGG groups at the class and family levels (*n* = 10–11).

## Supplementary figure 3

Functional profiles were obtained through Tax4Fun based on a KEGG database. The major identified pathways of each group rats were related to membrane transport, translation, replication and repair, carbohydrate metabolism, and amino acid metabolism (Figure S3A). But differences in predicted functional profiles of microbiota between each group rats were not significant (Figure S3B).

A

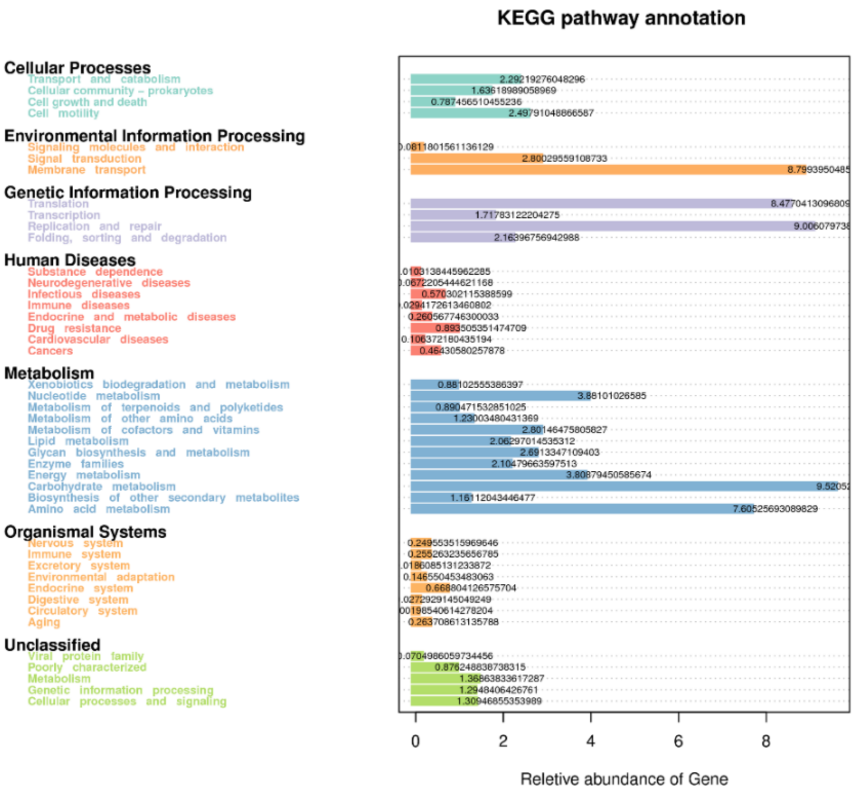


B

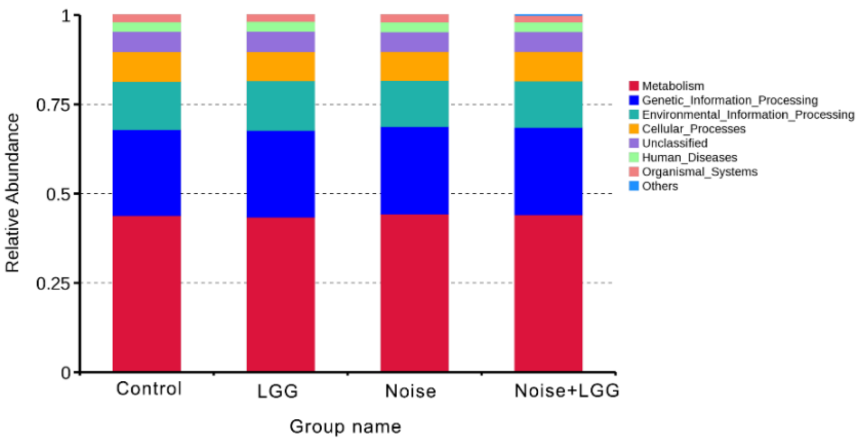


**Supplementary figure 3. Prediction of functional of gut microbiota of each group rats.** (A) KEGG pathway annotation and relative abundance of genes. (B) Predicted functional profiles of microbiota between each group rats.

**Experimental process of NDA extraction by CTAB method**

1. Absorb the 1000ulCTAB lysate into the 2.0mlEP tube, add 20ul lysozyme, add an appropriate amount of sample to the lysate, 65 degrees water bath (for fecal samples, water bath time 2 hours), reverse and mix several times during the period to make the sample fully split.

2. The supernatant of 950ul was centrifuged, and the same volume of phenol (PH8.0): chloroform: isoamyl alcohol (25:24:1) was added to the supernatant, and mixed upside down. 12000rpm centrifugation 10min.

3. Take the supernatant, add the same volume of chloroform: isoamyl alcohol (24:1), mix upside down, 12000rpm centrifugal 10min.

4. Absorb the supernatant into the 1.5mL centrifuge tube, add the supernatant 3 to 4 volumes of isopropanol, shake up and down, −20 degrees precipitate.

5. 12000rpm centrifuge for 10 minutes, pour out the liquid, be careful not to pour out the precipitation. Wash twice with 1ml 75% ethanol, the remaining small amount of liquid can be centrifuged again, and then sucked out with the gun head.

6. Super-clean table air-drying or room temperature drying.

7. Add 51 μ L ddH2O to dissolve DNA samples and incubate 10min at 55-60 °C if necessary.

8. Add RNase A 1ul to digest RNA, 37 °C and place 15min.
